# Novel Compounds Target Aberrant Calcium Signaling in the Treatment of Relapsed High-Risk Neuroblastoma

**DOI:** 10.3390/ijms26073180

**Published:** 2025-03-29

**Authors:** Dana-Lynn T. Koomoa, Nathan Sunada, Italo Espinoza-Fuenzalida, Dustin Tacdol, Madeleine Shackleford, Li Feng, Dianqing Sun, Ingo Lange

**Affiliations:** 1 College of Pharmacy and Health Sciences, Western New England University, Springfield, MA 01119, USA; 2 The Daniel K. Inouye College of Pharmacy, University of Hawaii at Hilo, Hilo, HI 96720, USAdianqing@hawaii.edu (D.S.)

**Keywords:** store-operated calcium entry (SOCE), high-risk neuroblastoma, mitochondria, endoplasmic reticulum, calcium release-activated calcium (CRAC) current (I^CRAC^), chemoresistance, apoptosis, proliferation, p53, MYCN

## Abstract

High-risk neuroblastoma (HRNB) is an extracranial solid pediatric cancer. Despite the plethora of treatments available for HRNB, up to 65% of patients are refractory or exhibit an initial response to treatment that transitions to therapy-resistant relapse, which is invariably fatal. A key feature that promotes HRNB progression is aberrant calcium (Ca^2+^) signaling. Ca^2+^ signaling is regulated by several druggable channel proteins, offering tremendous therapeutic potential. Unfortunately, many of the Ca^2+^ channels in HRNB also perform fundamental functions in normal healthy cells, hence targeting them increases the potential for adverse effects. To overcome this challenge, we sought to identify novel Ca^2+^ signaling pathways that are observed in HRNB but not normal non-cancerous cells with the hypothesis that these novel pathways may serve as potential therapeutic targets. One Ca^2+^ signaling pathway that is deregulated in HRNB is store-operated Ca^2+^ entry (SOCE). SOCE relays the release of Ca^2+^ from the endoplasmic reticulum (ER) and Ca^2+^ influx via the plasma membrane and promotes cancer drug resistance by regulating transcriptional programming and the induction of mitochondrial Ca^2+^ (mtCa^2+^)-dependent signaling. mtCa^2+^ signaling is critical for cellular metabolism, reactive oxygen production, cell cycle, and proliferation and has a key role in the regulation of cell death. Therefore, a dynamic interplay between ER, SOCE, and mitochondria tightly regulates cell survival and apoptosis. From a library of synthesized novel molecules, we identified two structurally related compounds that uniquely disrupt the dynamic interplay between SOCE, ER, and mitochondrial signaling pathways and induce cell death in HRNB. Our results revealed that compounds **248** and **249** activate distinct aberrant Ca^2+^ signals that are unique to relapsed HRNB and could be exploited to induce mtCa^+^ overload, a novel calcium influx current, and subsequent cell death. These findings establish a potential new pathway of calcium-mediated cell death; targeting this pathway could be critical for the treatment of refractory and relapsed HRNB.

## 1. Introduction

High-risk neuroblastoma (HRNB) is an extracranial solid tumor that has one of the least favorable outcomes of all pediatric cancers [1]. Despite the plethora of treatment options available for HRNB, including molecularly targeted therapy, immunotherapy, and multimodal treatments [2,3,4], up to 65% of patients are unresponsive (refractory) or exhibit an initial response to treatments that inevitably transitions to therapy-resistant relapse [5]. Finding an effective treatment strategy for patients with HRNB, particularly relapsed HRNB, continues to be a major challenge due to the aggressive phenotypes of these tumors and the myriad of complex mechanisms that promote resistance to treatments and recurrence. To develop more effective treatment strategies for this devastating disease, it is critical to elucidate the underlying signaling anomalies that drive therapy resistance and relapse in HRNB.

A key feature that promotes HRNB progression is aberrant calcium (Ca^2+^) signaling [6,7,8]. Ca^2+^ signaling is regulated by several druggable channel proteins, which offer tremendous therapeutic potential. Unfortunately, due to the fact that many of the Ca^2+^ channels in HRNB also perform fundamental functions in normal healthy cells, targeting them increases the potential for adverse effects [8,9]. To overcome this challenge, we sought to identify novel Ca^2+^ signaling pathways that are observed in HRNB but not normal non-cancerous cells with the hypothesis that these novel pathways may serve as potential therapeutic targets.

One Ca^2+^ signaling pathway that is upregulated in HRNB is the store-operated calcium entry (SOCE) [7,8]. SOCE links the release of Ca^2+^ from the endoplasmic reticulum (ER) to Ca^2+^ influx via ion channels in the plasma membrane. The stimulation of G-protein coupled receptors activates the formation of inositol 1,4,5-trisphosphate (IP3) via Phospholipase C, which opens IP3 receptors (IP3Rs) in the ER membrane, causing Ca^2+^ release into the cytosolic space. The depletion of ER Ca^2+^ transmits a signal from proteins that reside in the ER membrane to plasma membrane channels. This signal opens plasma membrane channels that allow high levels of extracellular Ca^2+^ to enter the cytosol and trigger subsequent Ca^2+^-dependent signaling [10,11]. SOCE promotes cancer drug resistance by regulating transcriptional programming [12,13,14,15]. Another consequence of increased cytosolic Ca^2+^ levels is the induction of mitochondrial Ca^2+^ (mtCa^2+^)-dependent signaling [16,17,18].

mtCa^2+^ signaling is essential for regulating a wide range of physiological processes, including cellular metabolism, reactive oxygen species (ROS) production, cell cycle progression, proliferation, autophagy, and the regulation of cell death [19,20].

The current study screened a library of novel compounds to identify compounds that disrupt the dynamic signaling between SOCE, ER, and mitochondrial pathways and induce cell death in HRNB. Our results found that compounds **248** and **249** activated aberrant Ca^2+^ signals that are unique to relapsed HRNB, which resulted in mtCa^+^ overload and cell death. These findings may lead to an effective treatment for Neuroblastoma (NB), particularly refractory and relapsed HRNB.

## 2. Results

### 2.1. Screening a Library of Synthesized Compounds

**Figure 1:**
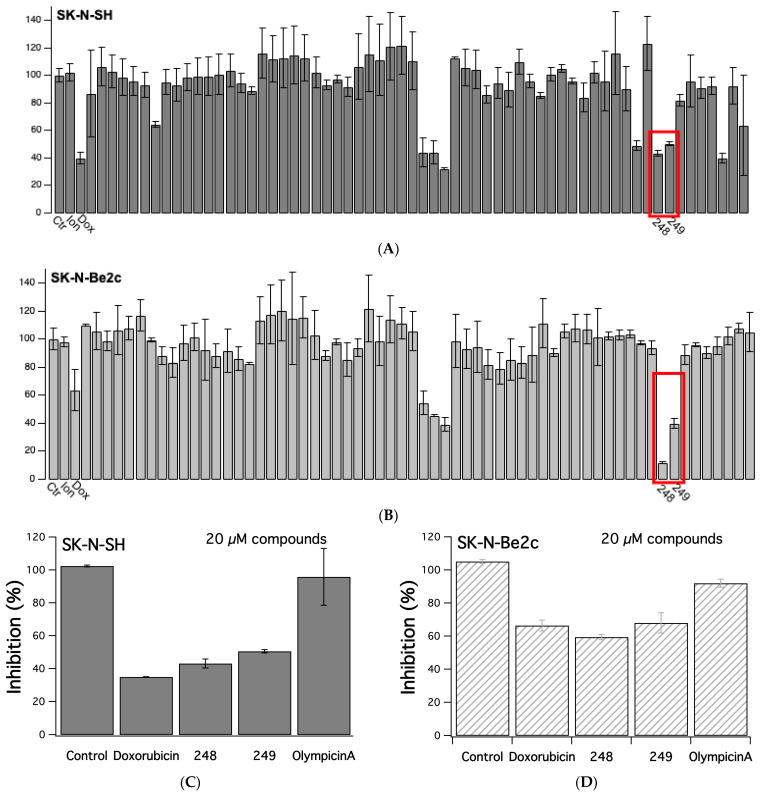
SRB cell viability screen of 62 compounds. SK-N-SH (**A**) and SK-N-Be2c (**B**) cells were incubated at 20 µM for 48 h. The y-axis depicts normalized cell viability in percentage. The red box shows the effects of **248** and **249** at 48 h. (**C**,**D**) show Control, 1 µM Ionomycin, and µM Doxorubicin and **248** and **249** in comparison to the parent compound Olympicin A.

A library of synthesized compounds was screened for anti-cancer properties in drug-sensitive and drug-resistant NB cells, SK-N-SH and SK-N-Be2(c), respectively. The sulfo-rhodamine B (SRB) assay was used to determine cell viability. NB cells were treated with 20 µM of each compound, vehicle (negative control), 1 µM ionomycin, or 1 µM doxorubicin (positive control) for 48 h. Out of the 62 compounds, eight showed potent anti-cancer effects in both SK-N-SH and SK-N-Be2(c) (Figure 1). Two of these compounds, **248** and **249**, had similar base structures and are analogs of Olympicin A. Compounds **248** and **249** decreased SK-N-SH cell viability by 58% and 53%, respectively, compared to control (vehicle) cells (Figure 1C), while doxorubicin decreased SK-N-SH cell viability by 65% (Figure 1C), compared to control cells. Compounds **248** and **249** decreased SK-N-Be2(c) cell viability by 43% and 36%, respectively, compared to control (vehicle) cells (Figure 1D), while doxorubicin decreased SK-N-Be2(c) cell viability by 37% (Figure 1D). Interestingly, Olympicin A (parent compound) had little to no effect on the viability of SK-N-SH and SK-N-Be2(c) cells (Figure 1C,D). The results show that the structurally related compounds **248** and **249** were able to effectively decrease NB cell viability.

### 2.2. Chemical Structures of Compound ***248*** and Compound ***249***

**Figure 2:**
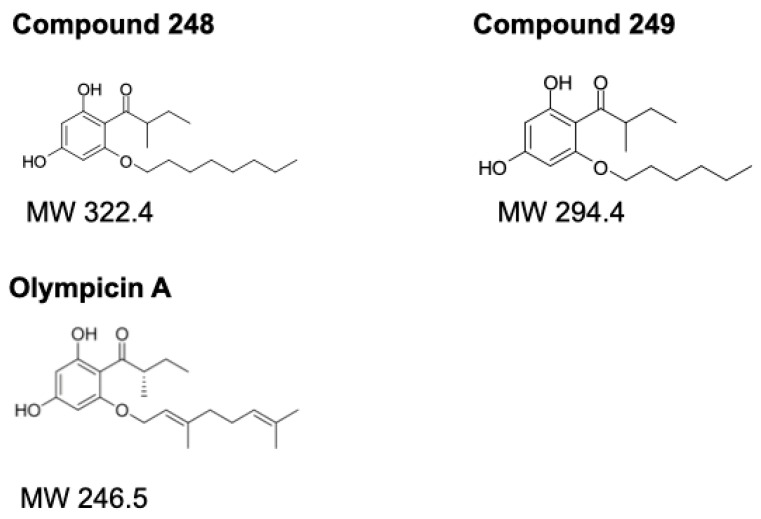
Structures and molecular weights of compound **248**, compound **249**, and Olympicin A.

Compounds **248** and **249** are analogs of Olympicin A, which is a member of the chemical class acylphloroglucinol. The structure of Olympicin A and compounds **248** and **249** are shown in Figure 2. Compounds **248** and **249** have the same base structure as Olympicin A; however, compounds **248** and **249** have (CH2)7-CH3 and (CH2)5-CH3 alkyl tails, respectively (Figure 2). The differences in the structures of compounds **248** and **249** may account for their enhanced anti-cancer properties compared to Olympicin A, which showed very little to no anti-cancer effects against NB cells.

### 2.3. Dose-Dependent Effects of Compound ***248*** and Compound ***249*** in NB

**Figure 3:**
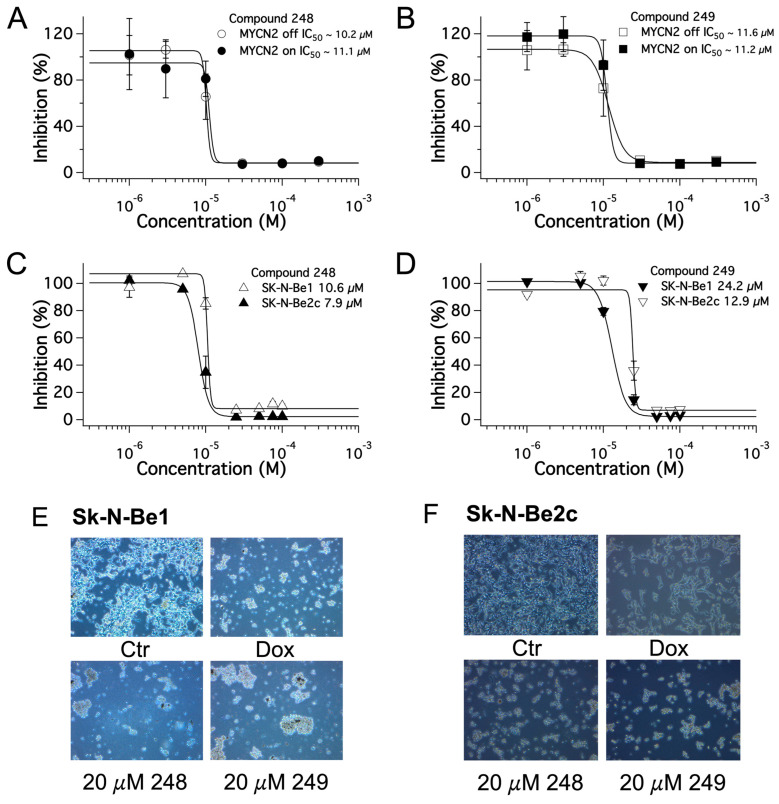
Establishing the IC50 concentrations of compounds **248** and **249** in neuroblastoma cells using cell viability assays. The dose-dependent effects of **248** and **249** on MYCN2 cells with and without MYCN overexpression (**A**,**B**) and on Sk-N-Be1 and SK-N-Be2c cells (**C**,**D**) determined using SRB assay. (**E**,**F**) Bright field acquisitions at 20 µM **248** and **249**. N ≥ 3.

Next, the dose-dependent effects of **248** and **249** on NB cell proliferation were examined. NB cells with tetracycline-inducible MYCN overexpression (MYCN2 +/− doxycycline), the drug-sensitive SK-N-Be1, and the drug-resistant SK-N-Be2(c) cells were used. Cells were treated with designated concentrations of **248** or **249** (from 0.1 μM to 3 mM), vehicle (negative control), or 1 μM doxorubicin (positive control) for 48 h. The SRB assay was used to determine NB cell viability at each designated concentration of **248** and **249**. The half-maximal inhibitory concentration (IC_50_) was then determined for **248** and **249**. The IC_50_ concentrations of **248** in MYCN2 cells with and without MYCN overexpression were 11.1 and 10.2 μM, respectively (Figure 3A), and the IC_50_ of compound **249** were 11.2 and 11.6 μM, respectively (Figure 3B). The IC_50_ concentrations of **248** in SK-N-Be1 and SK-N-Be2(c) were 10.6 and 7.9 μM, respectively (Figure 3C), and the IC_50_ of **249** were 24.2 and 12.9 μM (Figure 3D). The results show that compounds **248** and **249** reduced NB cell viability in a dose-dependent manner.

### 2.4. Compounds ***248*** and ***249*** Decrease Cell Cycle Proteins and Increase Apoptosis Markers

**Figure 4:**
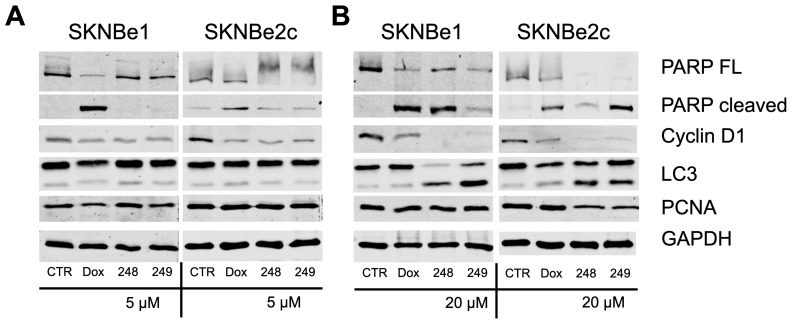
Dose-dependent effects of compounds **248** and **249** on apoptosis, DNA repair, cell cycle progression, autophagy, and cell proliferation. Western blot analysis of whole cell lysates prepared from neuroblastoma cells, Sk-N-Be1 and SK-N-Be2c, treated with 5 µM (**A**) or 20 µM (**B**) of compounds **248** and **249**. All experiments were performed in triplicate.

The effects of compounds **248** and **249** on cell cycle and apoptosis were further investigated by Western blot. NB cells treated with 5 μM 248, 20 μM 248, 5 μM **249**, 20 μM **249**, 1 μM doxorubicin, negative control (vehicle), and whole cell lysates were prepared after 48 h. The proteins in the lysate were separated by SDS-PAGE and transferred onto PVDF (Polyvinylidene Fluoride) membranes, and changes in the expression of proliferation, apoptosis, and autophagy markers were determined. For 1 μM doxorubicin, 5 μM **248** and **249**, and control untreated SK-N-Be1 cells, there were no changes in LC3-II (lipidated form of LC3-I (microtubule-associated protein 1A/1B-light chain 3), which is a marker of autophagy) and pCNA expression. In SK-N-Be1 cells, 1 μM doxorubicin, 5 μM **248**, and 5 μM **249** treatment decreased Cyclin D1 (marker of cell proliferation) expression compared to control untreated SK-N-Be1 cells. In SK-N-Be1 cells, 1 μM doxorubicin induced PARP cleavage (Poly (ADP-ribose) Polymerase, a marker for apoptosis) compared to the control (Figure 4A, left panel).

Additionally, 1 μM doxorubicin induced PARP cleavage and decreased Cyclin D1 expression in both SK-N-Be1 and SK-N-Be2c cells and LC3-II expression in only SK-N-Be2c cells compared to control untreated cells. In SK-N-Be1 and SK-N-Be2c cells, 20 μM **248** and **249** increased PARP cleavage, decreased CyclinD1 expression, and increased LC3-II expression compared to control untreated cells. Interestingly, pCNA expression decreased with 20 μM **248** and **249** in both SK-N-Be1 and SK-N-Be2c cells compared to the control (Figure 4A, right panel). The results show that **248** and **249** caused changes in the expression of proteins involved in proliferation, apoptosis, and autophagy in NB cells.

### 2.5. Compounds ***248*** and ***249*** Induce Ca^2+^ Signaling and Loss of Mitochondrial Membrane Potential

**Figure 5:**
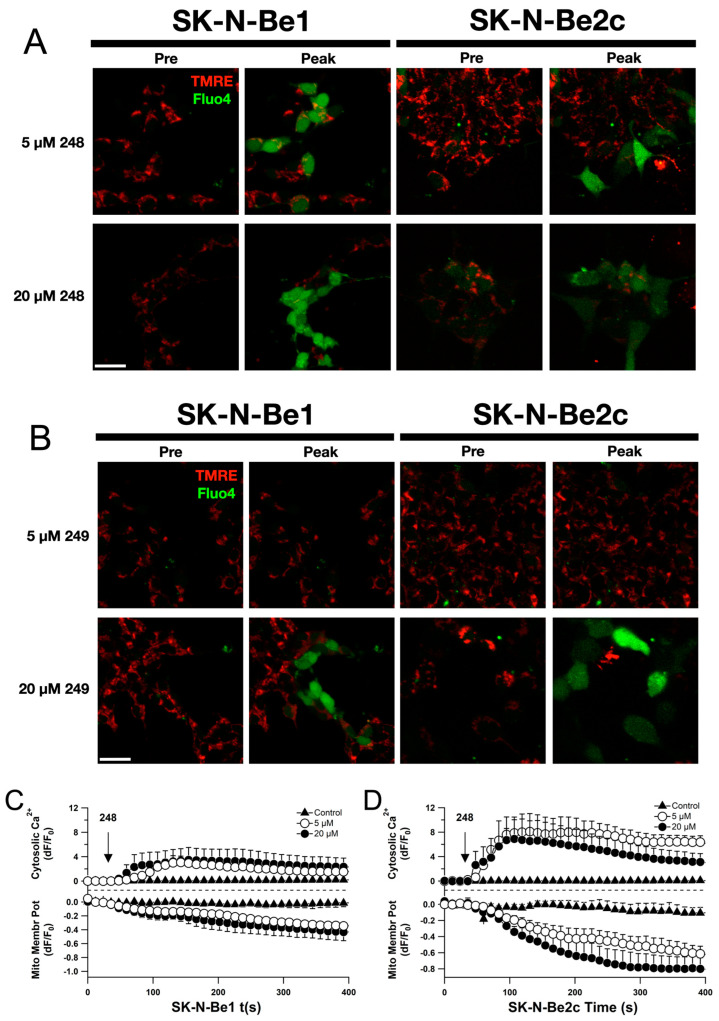

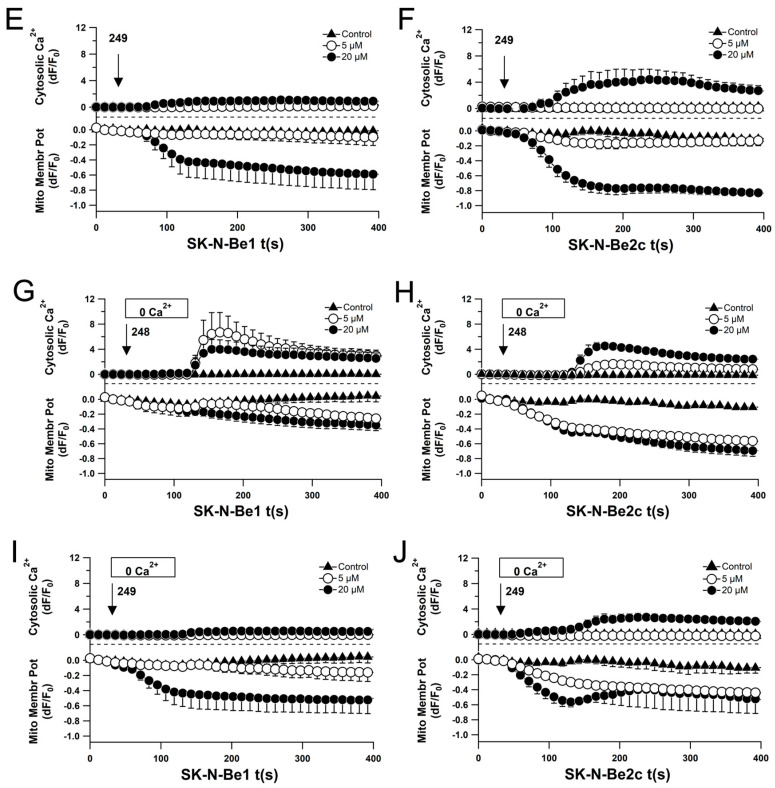
Effects of compounds **248** and **249** on disrupting the mitochondrial membrane potential and mobilizing cytosolic calcium in paired drug-sensitive and resistant NB cell lines. (**A**,**B**) Quantified signal of mitochondrial membrane potential and cytosolic calcium levels over time in response to different concentrations of **248** and **249** for Sk-N-Be1 and SK-N-Be2c cells in the presence (**C**–**F**) and absence (**G**–**J**) of external calcium. All experiments were performed in triplicate (n = 3); Scale bar (**A**,**B**) = 25 μm.

Next, the effects of compounds **248** and **249** on intracellular free calcium levels and mitochondrial membrane potential were determined. NB cells were loaded with Fluo-4 and TMRE (Tetramethylrhodamine Ethyl Ester, a fluorescent dye for mitochondrial membrane potential measurement), and time-lapsed imaging experiments were performed using a laser scanning confocal microscope. The results showed that in SK-N-Be1 cells, the intracellular free calcium levels increased 4-fold with 5 μM and 20 μM of compound **248**, respectively (Figure 5A,E). In SK-N-Be2(c), the intracellular calcium levels increased 7-fold and 6-fold with 5 μM and 20 μM of compound **248**, respectively (Figure 5B,F). The mitochondrial membrane potential decreased 4-fold with 5 μM and 20 μM of compound **248**, respectively, in SK-N-Be1 cells (Figure 5A,E). However, in SK-N-Be2(c), the mitochondrial membrane potential decreased 8-fold and 6-fold with 5 μM and 20 μM of compound **248**, respectively (Figure 5B,F).

In SK-N-Be1 cells, the intracellular free calcium levels increased 6-fold with 20 μM of compound **249** (Figure 5C,G). In SK-N-Be2(c), the intracellular calcium levels increased 5-fold with 20 μM of compound **249** (Figure 5B,F). The mitochondrial membrane potential decreased 6-fold with 20 μM of compound **249** in SK-N-Be1 cells (Figure 5C,G); however, in SK-N-Be2(c) cells, the mitochondrial membrane potential decreased 8-fold with 20 μM of compound **249** (Figure 5D,H). The results show that compounds **248** and **249** induced calcium signaling and loss of mitochondrial membrane potential in both SK-N-Be1 and SK-N-Be2(c) cells; however, compound **248** was more effective and induced larger calcium signals and greater reductions in mitochondrial membrane potential compared with compound **249**.

**Figure 6:**
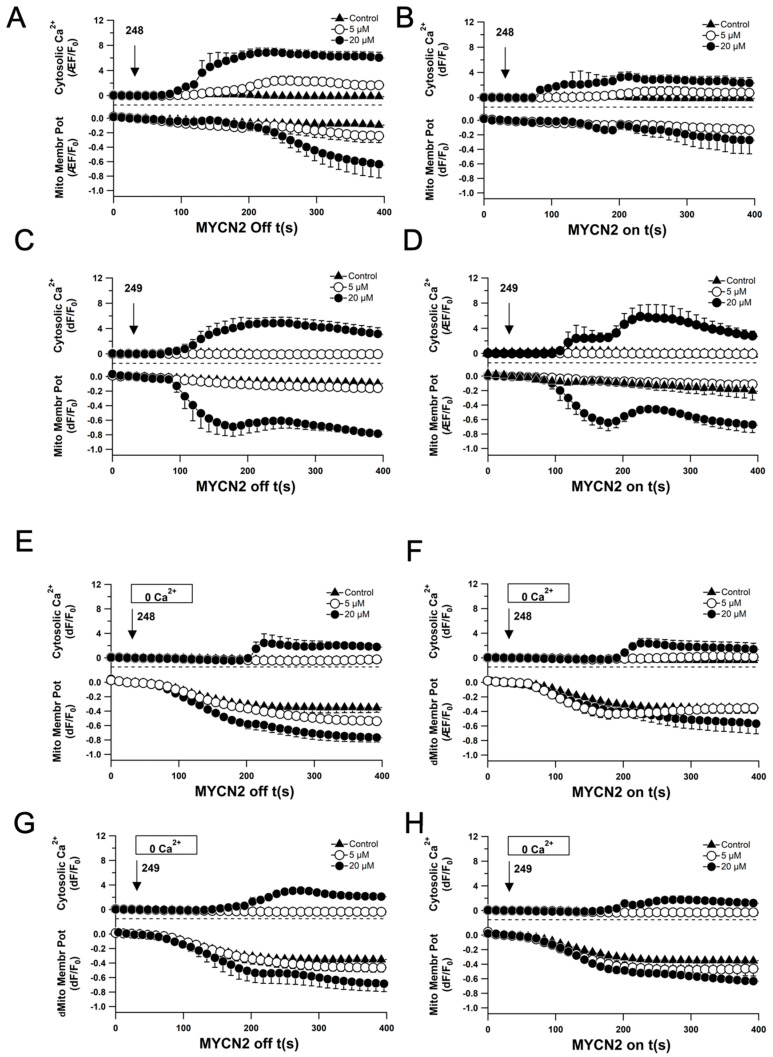
Effects of compounds **248** and **249** on disrupting the mitochondrial membrane potential and mobilizing cytosolic calcium in a model NB cell line of MYCN amplification. (**A**–**D**) Quantified signals of mitochondrial membrane potential and cytosolic calcium levels over time in response to different concentrations of **248** and **249** for confocal MYCN2 cells with and without MYCN overexpression in the presence (**A**–**D**) and absence (**E**–**H**) of external calcium. All experiments were performed in triplicate (n = 3).

Similar experiments were performed in the absence of extracellular calcium. Interestingly, mitochondrial membrane potential was not altered; however, the increase in cytosolic calcium levels was delayed, indicating the mobilization of intracellularly stored calcium in the cytoplasm. Overall, the ability of compound **249** to mobilize extracellular and intracellular calcium was decreased compared to that of compound **248**. The same set of experiments was performed using the MYCN2 cell line with and without overexpression of MYCN (Figure 6A–H). Similar results were obtained, with delayed cytosolic calcium increase when extracellular calcium was omitted; however, both compounds were comparably able to elicit an increase in extracellular calcium.

### 2.6. Compounds ***248*** and ***249*** Promote ER Calcium Release and Mitochondrial Calcium Uptake

**Figure 7:**
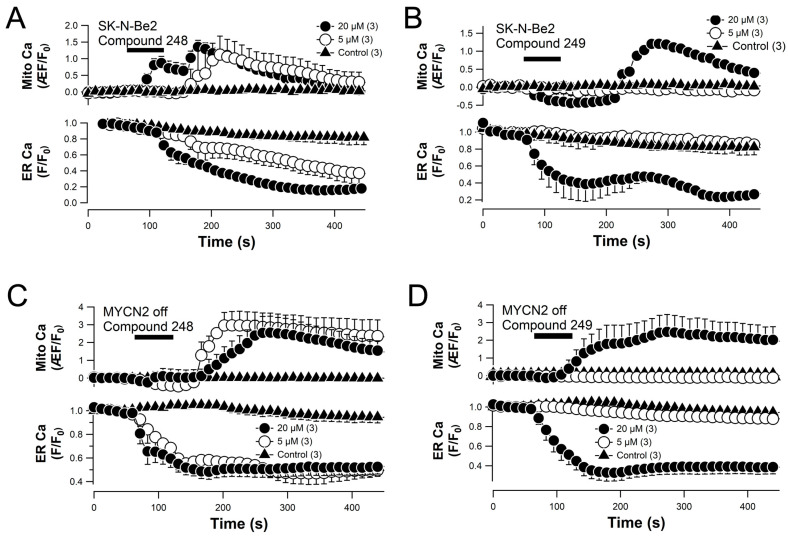

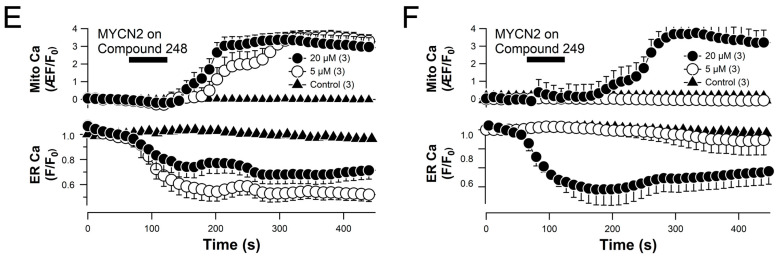
Compounds **248** and **249** mobilize mitochondrial and endoplasmic reticulum calcium using genetically engineered calcium indicators. SK-N-Be2c cells (**A**,**B**) and MYCN2 cells with (**C**,**D**) and without (**E**,**F**) MYCN overexpression were co-transfected with genetically engineered calcium indicators for mitochondrial (Mito Ca) and endoplasmic reticulum (ER Ca) calcium. (**A**–**F**) Quantified confocal signals of mitochondrial and endoplasmic reticulum calcium levels over time in response to different concentrations of **248** and **249** in the presence of external calcium. All experiments were performed in triplicate (n = 3); Back bar indicates time of compound application.

Genetically encoded fluorescent Ca^2+^ indicators targeted to the ER (pCMV G-CEPIA1er) [21] and the mitochondria (CMV-mito-R-GECO1) [22] were used to determine the effects of compounds **248** and **249** on ER and mitochondrial Ca^2+^ signaling in SK-N-Be2(c) cells. The results show that 5 μM and 20 μM of compound **248** increased mtCa^2+^ levels 1.1-fold and 1.3-fold, respectively (Figure 7A), and reduced ER calcium levels by ~60% and 85%, respectively (Figure 7A). Also, 20 μM of compound **249** increased mtCa^2+^ 1.5-fold (Figure 7B) and reduced ER calcium levels by ~80% (Figure 7B). The results show that compounds **248** and **249** induce ER calcium release and mtCa^2+^ uptake; however, compound **248** is more effective at inducing these changes in ER and mitochondrial calcium signaling. Similar patterns of calcium mobilization were observed for both compounds **248** and **249** when the experiments were performed using MYCN2 cells with and without overexpression of MYCN (Figure 7C–F).

### 2.7. Compounds ***248*** and ***249*** Activate Store-Operated Calcium Entry

**Figure 8:**
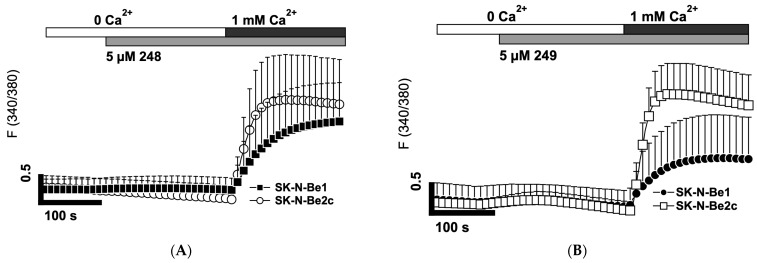

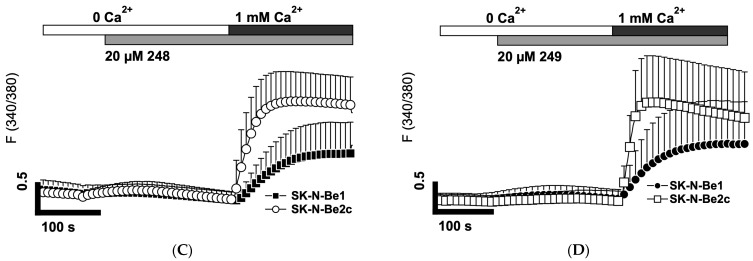
Compounds **248** and **249** induce store-operated calcium entry in NB cells. Cytosolic Ca^2+^ measurements using Fura-2 in Sk-N-Be1 and SK-N-Be2c cells. Ratiometric traces show 5 and 20 μM of **248** and **249** activating Ca^2+^ release in nominally Ca^2+^-free bath solutions and SOCE when extracellular Ca^2+^ was restored. Compounds perfused cells in the absence of calcium to resolve intracellular store depletion. Subsequent readmission of extracellular calcium indicates plasma-membrane mediated store-operated calcium influx (**A**–**D**). All experiments were performed n = 3.

Due to the ER calcium release induced by the treatment of NB cells with compounds **248** and **249**, the effects of these compounds on the induction of store-operated calcium entry were examined. Classical experiments to study SOCE using Fura-2 am were performed. Compounds **248** and **249** (5 μM) in the absence of extracellular calcium provoked an increase in intracellular calcium, returning to basal levels after a duration of ~300 s, indicating calcium mobilization out of stores until fully depleted. Re-admission of extracellular calcium rapidly elicited an influx of cytosolic calcium through the plasma membrane (Figure 8A,B). SOCE was reduced in the SK-N-Be1 cells compared to the SK-N-Be2c cells, indicating fewer plasma membrane channels. When the concentrations of compounds **248** and **249** were increased to 20 μM, similar patterns and intensities were observed (Figure 8C,D).

### 2.8. Compounds ***248*** and ***249*** Activate Store-Operated Calcium Entry Resembling a Current That Is Characteristic of ORAI Channels

**Figure 9:**
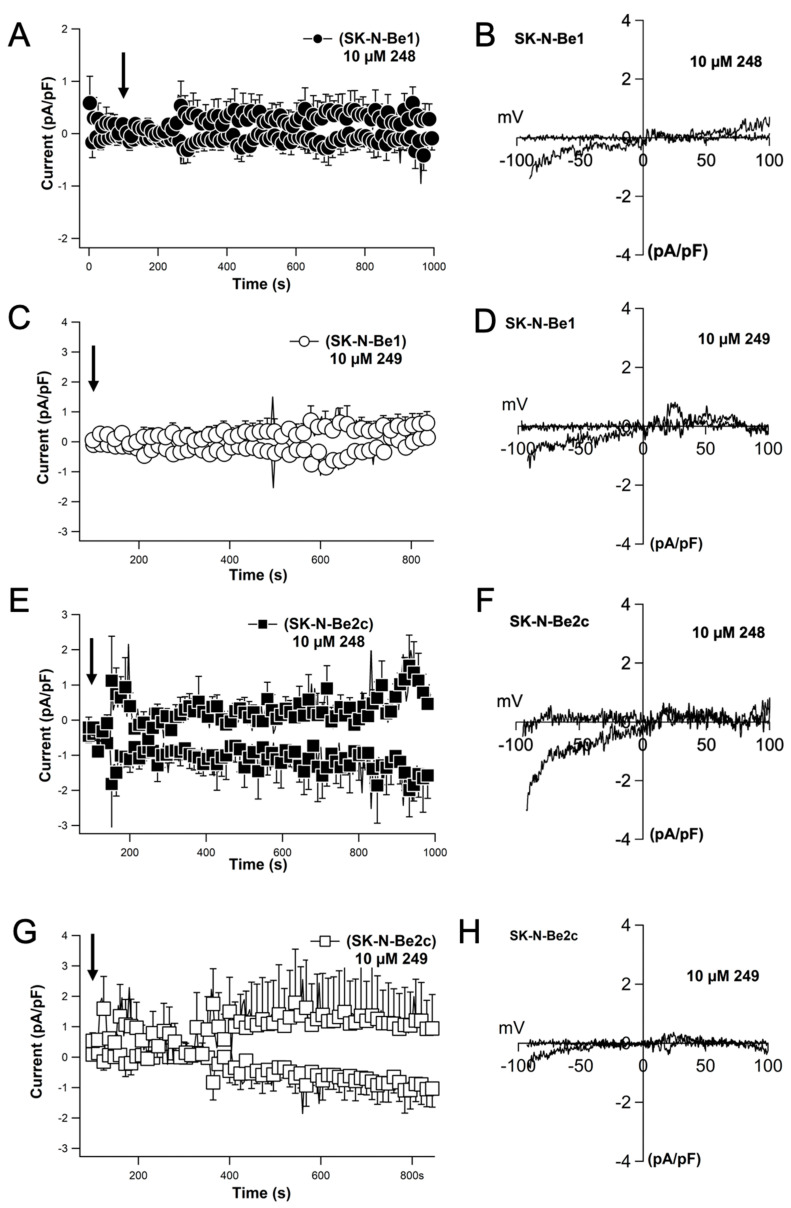
Compounds **248** and **249** (10 µM) induce current–voltage relationship characteristics for currents mediated by ORAI. Currents activated by extracellular application of 10 µM of **248** and **249**. Current amplitudes are measured at −80 mV and +80 mV, normalized for cell size, and averaged (**A**,**C**,**E**,**G**). Time course of current development at −80 and +80 mV induced by extracellular perfusion of 10 µM of **248** and **249** in Sk-N-Be1 and SK-N-Be2c cells (**A**,**C**,**E**,**G**). Representative current–voltage relationship obtained by a 100 ms voltage ramp pulse (−100 mV to + 100 mV) (**B**,**D**,**F**,**H**). Data points are means ± S.E.M. The arrow indicates the beginning of extracellular application of compounds.

Due to the calcium influx pattern evoked by compounds **248** and **249**, which is characteristic of store-operated calcium, electrophysiological experiments were performed to understand the nature of the currents evoked. Whole-cell configuration of electrophysiological patch-clamp measurements were recorded in SK-N-Be2(c) cells. The store-operated calcium current (I_crac_) density was determined at +80 mV and −80 mV. The external application of 10 μM of compound **248** increased the current density and produced a very small inward current of approximately 1 pA/pF in SK-N-Be1 cells (Figure 9A). The current–voltage (I/V) relationship resembled that of I_crac_, the current observed with store-operated calcium entry as mediated by ORAI channels (Figure 9B). The external application of 10 μM of compound **248** increased the current density and produced a larger inward current of approximately 3 pA/pF in SK-N-Be2(c) cells (Figure 9E) and the current–voltage (I/V) relationship characteristic of I_crac_ (Figure 9F). Similar results were obtained with 10 μM of compound **249** (Figure 9C,D,G,H). However, the increase in current density observed and the I/V curve with 10 μM of compound **249** (Figure 9C,D,G,H) were less than that of compound **248** (Figure 9A,B,E,F) in both SK-N-Be1 and SK-N-Be2(c) cells. The results indicate that compounds **248** and **249** induce I_crac_. However, compound **248** was more effective at activating the current than compound **249**. Compounds **248** and **249** at concentrations that initiate cell death induce an unidentified nonlinear current.

**Figure 10:**
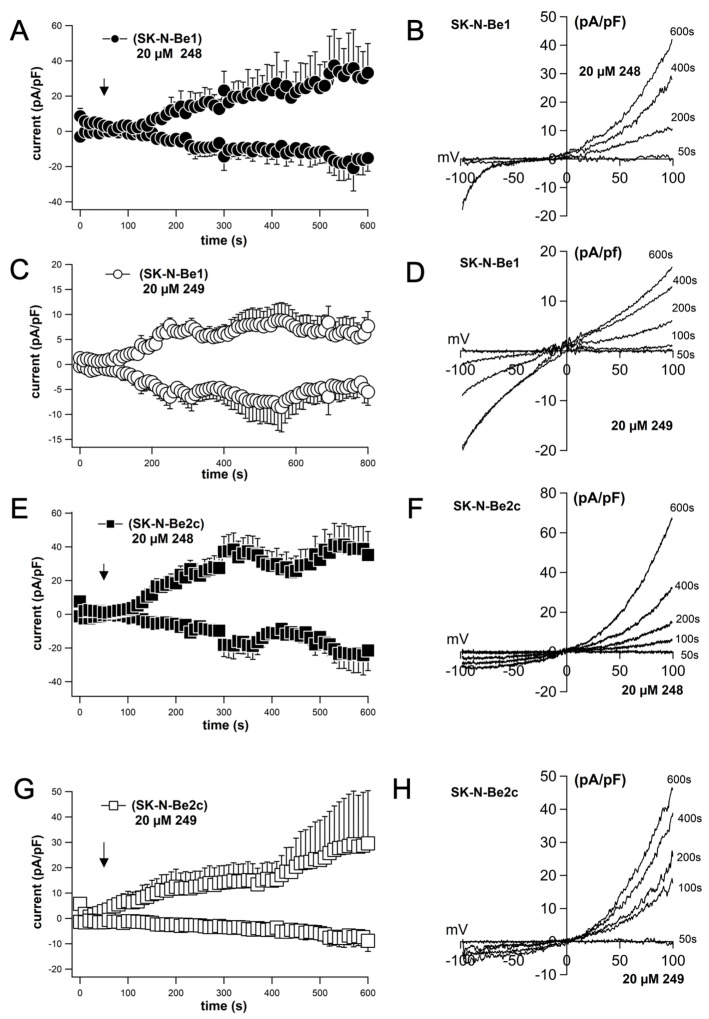
Compounds **248** and **249** (20 µM) induce a non-linear current–voltage relationship. Currents activated by extracellular application of 20 µM of **248** and **249**. Current amplitudes are measured at −80 mV and +80 mV, normalized for cell size, and averaged (**A**,**C**,**E**,**G**). Time course of current development at −80 and +80 mV induced by extracellular perfusion of 20 µM of **248** and **249** in Sk-N-Be1 and SK-N-Be2c cells (**A**,**C**,**E**,**G**). Representative current–voltage relationship obtained by a 100 ms voltage ramp pulse (−100 mV to + 100 mV) (**B**,**D**,**F**,**H**). Data points are means ± S.E.M.

**Figure 11:**
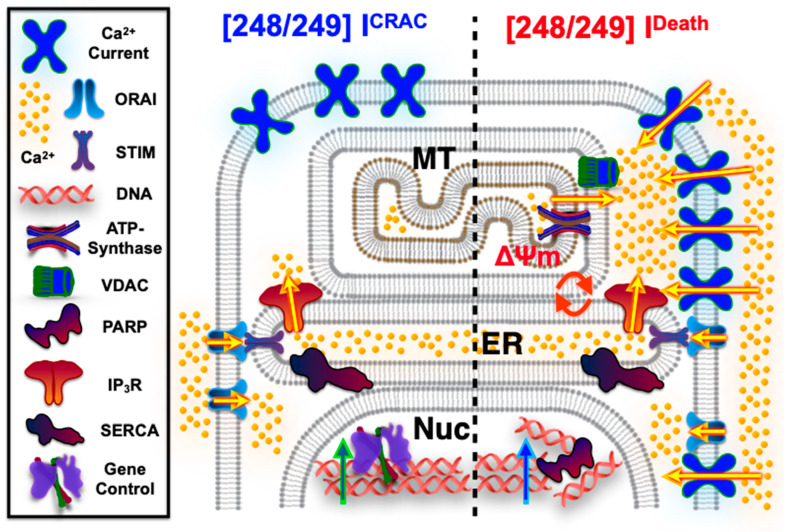
Graphical presentation of the 2 distinct pathways that compounds **248** and **249** induce in a dose-dependent manner. The left side of the schematic, separated by a dotted line, illustrates the scenario where lower concentrations of compounds **248** and **249** deplete endoplasmic reticulum (ER) calcium stores, activate the Calcium Release-Activated Calcium (CRAC) current (I^CRAC^) via ORAI, and subsequently regulate nuclear (Nuc) gene expression. The right side depicts the scenario where higher concentrations of compounds **248** and **249** mobilize calcium from both the ER and mitochondria (MT), disrupt mitochondrial membrane potential (ΔΨm), and induce a calcium influx current (Ca^2+^ Current) of unknown molecular identity, leading to apoptotic signaling through PARP activation. Arrows indicated flow of calcium (golden), activation of gene regulation (green) or PARP action (blue).

Compounds **248** and **249** induce cell death in a dose-dependent manner; however, the potency they elicit is similar, as seen in drugs with a narrow therapeutic index indicated by the steepness of the slope as observed in Figure 3A–C. To investigate whether an increased concentration of the compounds would alter the plasma membrane conductance, we performed patch-clamp experiments using the same conditions as in previously applied whole-cell patch-clamp experiments in Figure 9; however, we used 20 μM of compounds **248** and **249**, which is more than the IC_50_ that induces cell death. Indeed, 20 μM of compounds **248** and **249** induced a large nonlinear current over the course of 300 s of application that was slightly outward rectified (Figure 10A–F). Overall, **248** induced slightly larger currents compared to **249** and also displayed greater currents in SK-N-Be2c cells compared to Sk-N-Be1 cells. This observation was similar to the cytosolic calcium levels evoked by **248** and **249** that were observed in the calcium imaging experiments (Figure 11).

## 3. Discussion

Up to 65% of NB patients are diagnosed with high-risk disease and are refractory to treatment or experience tumor recurrence. Despite the plethora of treatment options, there are no effective treatments for high-risk relapsed NB, which is inevitably fatal. The development of more effective treatment options remains a major clinical challenge in the treatment of NB. The current study focused on identifying novel compounds that induce cell death in high-risk relapsed NB that may potentially enhance treatment options for patients with this fatal disease.

A library of 60 synthesized compounds was screened for anti-cancer properties in SK-N-SH and SK-N-Be2(c) NB cells. SK-N-SH are NB cells with non-amplified MYCN2 gene. SK-N-Be2(c) NB cells are tumor cells excised from a patient after relapse (drug resistant). Two compounds, **248** and **249**, exhibited potent anti-cancer effects. The dose-dependent effects of these compounds were examined in the following NB cell lines: (1) MYCN2: NB cells with and without doxycycline-inducible MYCN overexpression; (2) SK-N-Be1: tumor cells derived from a patient at diagnosis (responsive to chemotherapy); (3) SK-N-Be2(c): tumor cells derived from the same patient after relapse (drug resistant). Table 1 shows the IC_50_ values for compounds **248** and **249** in these cells. Interestingly, the effects of **248** and **249** were similar in MYCN2 cells with and without the overexpression of MYCN. The effects of **248** and **249** on NB cell viability were comparable to that of doxorubicin, a drug that is used to treat NB patients; however, **248** and **249** were more effective in the SK-N-Be2(c) cells compared to the SK-N-Be1 cells. In addition, **248** was more effective at reducing NB cell viability than **249**; therefore, while both compounds reduced the viability of all NB cells tested, they were both more effective in the drug-resistant SK-N-Be2(c) cells than in the other NB cell types. These effects appear to be independent of MYCN.

*MYCN* gene amplification is a prognostic indicator in high-risk NB. The MYCN protein is a transcription factor that plays a key role in regulating the expression of many genes that promote NB progression, including, but not limited to, Ornithine Decarboxylase (ODC1), a rate-limiting enzyme in the biosynthesis of polyamines, as well as MDM2, TP53, Skp2, and PI3K/Akt/mTOR. Interestingly, amplification of the *MYCN* gene and overexpression of the MYCN protein did not appear to influence the effect of compound **248**, as the IC_50_ for this compound was similar in MYCN2 cells regardless of MYCN overexpression, as well as in SK-N-Be1 cells, which have been characterized with amplification of the *MYCN* gene. Interestingly, the IC_50_ for **248** was much lower in SK-N-Be2(c) cells, which also exhibit amplification of the *MYCN* gene. In addition, **249** had similar effects in MYCN2 cells with and without MYCN overexpression. The effects of **249** were less pronounced in SK-N-Be1 cells; however, SK-N-Be2(c) cells were more sensitive to **249** than SK-N-Be1 cells. Therefore, the results indicate that the anti-cancer effects of **248** and **249** are not influenced by *MYCN* gene amplification or the overexpression of the MYCN protein. The different effects of the compounds in the different cells may be associated with other factors.

TP53 is a tumor suppressor gene, and the p53 protein product performs many functions, including, but not limited to, DNA repair, regulating cell cycle progression and inducing apoptosis. It is well documented that TP53 is mutated in many types of cancers [23]. Wild-type p53 is expressed in MYCN2 cells and SK-N-Be1 cells [24]. However, there is a p53 missense mutation in SK-N-Be2(c) cells, with a substitution of cysteine 135 for phenylalanine [25]. Interestingly, the IC_50_ of **248** was similar in MYCN2 cells with and without MYCN overexpression and in SK-N-Be1 cells. However, The IC_50_ of **248** was much lower in SK-N-Be2(c) cells compared to the other cell types tested. In addition, the IC_50_ of **249** was similar in MYCN 2 cells with and without MYCN overexpression, while the IC_50_ of **249** was higher in SK-N-Be1 cells compared to MYCN2 cells with and without MYCN overexpression, and the IC_50_ of **249** was significantly lower in SK-N-Be2(c) cells compared to SK-N-Be1 cells. The results suggest that p53 may influence the anti-tumor effects of **248** and **249** in NB, as p53 is mutated in SK-N-Be2(c), which could account for the enhanced anti-tumor effects of the compounds in SK-N-Be2(c) compared to the other NB cell types tested. However, this does not rule out other signaling pathways that may be involved in the mechanism of action of these compounds.

Previous studies have shown that *MYCN* gene amplification and MYCN overexpression increase calcium signaling in NB cells [7]. Numerous studies have identified calcium channel proteins and channel regulators that play a role in NB progression [8]. Therefore, the changes in Ca^2+^ signaling induced by **248** and **249** were examined. As presented in the Results Section, changes in Ca^2+^ influx, ER Ca^2+^, and mtCa^2+^ were measured over time. As mentioned above, to record the latter two calcium measurements, the NB cells were double transfected with genetically encoded fluorescent Ca^2+^ indicators targeted to the ER (pCMV G-CEPIA1er) and the mitochondria (CMV-mito-R-GECO1). However, due to the very slow doubling time of Be1, only MYCN2 and Be2c were successfully double transfected. In addition, the effects of **248** and **249** on mitochondrial membrane potential were measured over time.

Compounds **248** (5 μM and 20 μM) and **249** (20 μM) induced changes in Ca^2+^ signaling via all three parameters (i.e., Ca^2+^ influx, ER Ca^2+^, and mtCa^2+^) measured in MYCN2 with and without MYCN overexpression and in SK-N-Be2(c) cells. The 5 μM concentration of compound **249** did not induce changes in the three parameters in any of the cell types tested. Similar results were observed with the effects of **248** and **249** on changes in mitochondrial membrane potential. The loss of ER Ca^2+^ and induction of Ca^2+^ influx was characteristic of store-operated calcium entry (SOCE). Whole-cell configuration patch-clamp electrophysiological recordings confirmed the activation of SOCE by **248** and **249**. The augmented SOCE may increase the driving force for mtCa^2+^. The increase in mtCa^2+^ and the change in mitochondrial membrane potential suggest that mtCa^2+^ overload may account for the loss of cell viability induced by **248** and **249**. Overall, **248** and **249** exhibited potent anti-cancer effects through a mechanism that may involve enhanced SOCE, which increases the driving force for mtCa^2+^ uptake and induces apoptosis despite the altered mtCa^2+^ signaling. These aberrant calcium signals in relapsed high-risk NB may account for the different effects of the compounds in different NB cells. The cytotoxic effect may be further enhanced by additional cytosolic calcium increase by the current that is evoked at concentrations over 20 μM for both **248** and **249**. The molecular identity and mechanism of activation of this current are currently unknown. Potential activation of plasma membrane current through calcium should be prevented as intracellular calcium concentrations are clamped to 150 nM by the use of 10 mM BAPTA, hence antagonizing calcium-activated calcium currents. The slope of the SRB assay is very steep, indicating that a separate event that contributes to drastic cell death occurs rapidly. Interestingly, 1–5 μM of **248** and **249** increased proliferation compared to control cells; however, increasing their concentrations to 20 μM led to rapid cell death, indicating that another separate event, possibly this unknown current (I^U^), may contribute to additional calcium overload and death. The higher IC_50_ value required to induce cell death in the chemo-sensitive cells may also be explained by the observation that SK-N-Be1 cells have overall less SOCE currents as well as less I_U_-current; hence, calcium overload is delayed or reduced, and cell death is achieved at higher concentrations of the compounds compared to the drug-resistant counterpart.

The greater efficacy of **248** compared to **249** prompted further analysis of these two compounds. Compounds **248** and **249** are analogs of the parent compound, Olympicin A, an acylphloroglucinol from *Hypericum olympicum*. Several different acylphloroglucinols have been shown to have anti-cancer effects. Compounds **248** and **249** have different functional groups than Olympicin A, exhibiting long alkyl chains (Figure 2). Compound **248** has R-(CH_2_)_7_-CH_3_ and compound **249** has R-(CH_2_)_5_-CH_3_. The alkyl chains may account for the greater anti-cancer effects of **248** and **249** compared to the parent compound, Olympicin A, which did not show a significant anti-cancer effect. In addition, the longer alkyl chain of **248** may account for the greater anti-cancer effects compared to **249**. Presumably, the alkyl chains allow the compounds to be more permeable to the cell membrane and increase the ability of these compounds to enter the cell. This logic would assume that **248** would be more cell-permeable than **249**, and both **248** and **249** are more cell-permeable than Olympicin A. Therefore, the structures of **248** and **249** play a role in the efficacy of the compounds as anti-cancer agents, and the increased efficacy of **248** compared to **249**.

In conclusion, the current study identified two novel compounds that are effective against NB, including relapsed high-risk NB. These compounds have a novel mechanism that involves augmented SOCE that increases the threshold for calcium uptake into the mitochondria. This increased driving force for mtCa^2+^ uptake overcomes the altered mtCa^2+^ transport machinery and leads to the induction of apoptosis (Figure 9). Beyond the IC_50_ levels of **248** and **249**, an unknown current is induced that may contribute to calcium overload and rapid cell death. These compounds may be useful tools for studying these calcium signaling pathways and may serve as alternative treatment options for relapsed high-risk NB.

## 4. Materials and Methods

### 4.1. Cell Culture

MYCN2 is a tetracycline-inducible MycN overexpression NB cell line (provided by Dr. Jason Shohet). SKNBe1 and SKNBe2c are NB cell lines derived from tumors removed from a patient at diagnosis and after relapse, respectively (Children’s Oncology Group). SK-N-SH cells (ATCC HTB-11) were purchased from the American Type Culture Collection (Manassas, VA, USA). The cells were cultured in RPMI-1640 (Mediatech, Inc., Manassas, VA, USA) supplemented with 10% (*v*/*v*) heat-inactivated fetal bovine serum (FBS) (Atlanta Biologicals, Lawrenceville, GA, USA) and grown at 37 °C, 5% CO_2_, and 95% humidity. All cells were authenticated by the cell line authentication testing services at Genetica DNA laboratories (Cincinnati, OH, USA) using STR DNA typing to verify each cell line and purity from contamination [6].

### 4.2. Chemicals

General chemicals were obtained from VWR (West Chester, PA, USA). Doxorubicin and ionomycin were obtained from Calbiochem (Gibbstown, NJ, USA).

### 4.3. Synthesis of Compounds

The compounds in the library were synthesized as described previously [26].

### 4.4. Changes in Intracellular Free Calcium and Mitochondrial Membrane Potential

Calcium levels and mitochondrial membrane potential (ΔΨm) were determined as previously described [6]. Briefly, NB cells were washed and incubated with 2 µM of the acetoxymethyl ester form of Fluo-4 (Molecular Probes, Eugene, OR, USA) for 30 min at 37 °C in standard modified Ringer’s solution of the following composition (in mM): NaCl 145, KCl 2.8, CsCl 10, CaCl_2_ 0–2, MgCl_2_ 0–2, glucose 10, Hepes·NaOH 10 (pH 7.4), 300–330 mOsm. TMRE (20 nM) was added and incubated for an additional 15 min at room temperature. For calcium-free experiments, 1 mM EGTA was added to the external solution, and calcium chloride was omitted. Cells were transferred to 96-well plates at 10,000 cells/well and stimulated as indicated. Confocal measurements were performed using a Leica SPE. Fluorescence intensity was quantified and analyzed using Leica LAS AF (Mannheim, Germany). For Fura-2 experiments, cells were loaded with 2.5 μM Fura-2-AM (acetoxymethylester, Invitrogen, Waltham, MA, USA) for 30 min at 37 °C. Time-lapse experiments were recorded at 0.5 Hz, 340/380 nm excitation, and 510 nm emission using a Horiba/Photon Technology International (Piscataway and Birmingham, NJ, USA) imaging system and 63X oil objective.

### 4.5. Time Lapsed Live-Cell Imaging

Genetically encoded fluorescent Ca^2+^ indicators targeted to the ER (pCMV G-CEPIA1er) and the mitochondria (CMV-mito-R-GECO1) were used to visualize changes in ER Ca^2+^ and mtCa^2+^ levels in real time. NB cells were transfected with the genetically encoded fluorescent Ca^2+^ indicators. NB cells were incubated and maintained in Ringer’s extracellular media (in mM: NaCl 140, KCl 2.8, Na-HEPES 10 (pH 7.2), CaCl_2_ 1, MgCl_2_ 1, Glucose 11, adjusted to 290 mOsm). Single-cell time-lapse recordings were acquired using a Leica SPE confocal microscope using a Leica 63X/1.40 oil immersion objective and appropriate excitation lasers and emission filters. Images were obtained every 3.96 s. A Picospritzer III micro-perfusion system was used (Parker Hannifin, Hollis, NH, USA) to apply different drug treatments using glass borosilicate micropipettes in a horizontal puller (DMZ-Universal puller, Zeitz, Martinsried, Germany) driven by a micromanipulator (MPC-200, Sutter Instruments, Novato, CA, USA). The time-lapse images were processed on Leica LAS AF (Leica Application Suite Advanced Fluorescence, Version 2.5.1.6757) software. The calcium levels were calculated and presented as changes in relative fluorescence over time, as published elsewhere [17]. The data were analyzed using Volocity software (PerkinElmer, Waltham, MA, USA) and averaged at each time point. Further calculations were carried out using a Python 3.2 script, and data were plotted using Igor Pro 6 (Wavemetrics, Portland, OR, USA).

### 4.6. Electrophysiology

Electrophysiological experiments were recorded as previously reported [6]. For patch-clamp experiments, cells were kept in standard external solution (in mM): 140 NaCl, 2.8 KCl, with 1-10 CaCl_2_, 2 MgCl_2_, 11 glucose, 10 HEPES·NaOH (pH 7.2 adjusted with NaOH, 300–320 mOsm). Standard pipette-filling solutions contained (in mM) 120 Cs-glutamate, 8 NaCl, 1 MgCl_2_, 10 HEPES|CsOH, 10 Cs-BAPTA (pH 7.2 adjusted with CsOH, 290–310 mOsm). Intercellular calcium was clamped to ~150 nm as calculated by WebMaxC (https://somapp.ucdmc.ucdavis.edu/pharmacology/bers/maxchelator/webmaxc/webmaxcS.htm, accessed on 17 March 2025).

### 4.7. Sulforhodamine B Assay

The SRB (*Sulforhodamine B)* colorimetric assay was used to determine cell proliferation following the protocol previously described [6]. Briefly, cells were seeded at a density of 10,000 cells/well on a transparent, flat-bottom, 96-well plate and allowed to settle overnight. At the initiation of each experiment (*t* = 0) and after drug treatments, 100 μL of 10% (*w*/*v*) TCA were added to each well, incubated for 1 h at 4 °C, washed with deionized water, and dried at room temperature. One hundred microliters of 0.057% (*w*/*v*) SRB solution were added to each well, incubated for 30 min at room temperature, rinsed four times with 1% (*v*/*v*) acetic acid, and allowed to dry at room temperature. Finally, 200 μL of 10 mM Tris base solution (pH 10.5) was added to each well, and after shaking for 5 min at room temperature, absorbance was measured at 510 nm in a microplate reader. The absorbance at *t* = 0 was compared with the absorbance at the end of the experiment to determine the growth of treated cells compared with that of control cells. All experiments were independently repeated a minimum of 3 times.

### 4.8. Western Blot Analysis

Cell lysates were prepared in radioimmunoprecipitation assay buffer [20 mmol/L Tris-HCl (pH 7.5), 0.1% (*w*/*v*) sodium lauryl sulfate, 0.5% (*w*/*v*) sodium deoxycholate, 135 mmol/L NaCl, 1% (*v*/*v*) Triton X-100, 10% (*v*/*v*) glycerol, 2 mmol/L EDTA] supplemented with Complete protease inhibitor cocktail (Roche Molecular Biochemicals, Basel, Switzerland) and phosphatase inhibitors sodium fluoride (20 mmol/L) and sodium vanadate (0.27 mmol/L). Western blot analysis was conducted as previously described [27]. The total protein concentration was determined using the protein assay dye reagent from Bio-Rad Laboratories (Hercules, CA, USA). Cell lysates in SDS-sample buffer were boiled for 5 min, and equal amounts of total protein were analyzed by 10% SDS-PAGE and Western blotting. The antibodies used in this study are rabbit polyclonal CyclinD1 (1:1000), rabbit polyclonal LC3 (1:1000), rabbit polyclonal cleaved PARP (1:1000), mouse monoclonal GAPDH (1:1000), and mouse monoclonal PCNA (1:1000) from Cell Signaling Technology. Proteins were detected using the Odyssey Infrared Imaging System (LI-COR Biosciences, Lincoln, NE, USA) and analyzed with Licor Image Studio 2.0 acquisition and analysis software.

### 4.9. Analysis

Results are shown as the mean ± standard deviation. Statistical significance was determined based on a two-way analysis of variance (Student’s *t*-test). Significant differences were recorded adjacent to data points in the respective graphs. Western blot, SRB, and Fluo-4 experiments were all conducted at least in triplicate (i.e., *n* = 3 or more independent experiments). Electrophysiological and calcium measurements were *n* = 5 or more. All other experiments are *n* = 3 or more independent experiments.

## Figures and Tables

**Table 1:** IC50 values for compound **248** and compound **249**.

	248 (µM)	249 (µM)
MYCN2 (− doxy)	10.2	11.6
MYCN2 (+ doxy)	11.1	11.2
SK-N-Be1	10.6	24.2
SK-N-Be2(c)	7.9	12.9

## Data Availability

The original contributions presented in this study are included in the article. Further inquiries can be directed to the corresponding authors.
